# 1-(4-Meth­oxy­phen­yl)-2-methyl-1*H*-indole-3-carbonitrile

**DOI:** 10.1107/S1600536811031035

**Published:** 2011-08-11

**Authors:** Qiao Yan, Xiuxiang Qi

**Affiliations:** a School of Pharmaceutical Science and Technology, Tianjin University, Tianjin 300072, People’s Republic of China

## Abstract

In the title compound, C_17_H_14_N_2_O, the dihedral angle between the indole ring system and the benzene ring is 58.41 (4)°. The crystal packing features π–π stacking [shortest centroid–centroid separation = 3.8040 (9) Å] and C—H⋯π inter­actions.

## Related literature

For the synthesis of the title compound, see: Du *et al.* (2006 ▶). For its precursor, see: Jin *et al.* (2009 ▶). For a related structure, see: Yang *et al.* (2011 ▶).
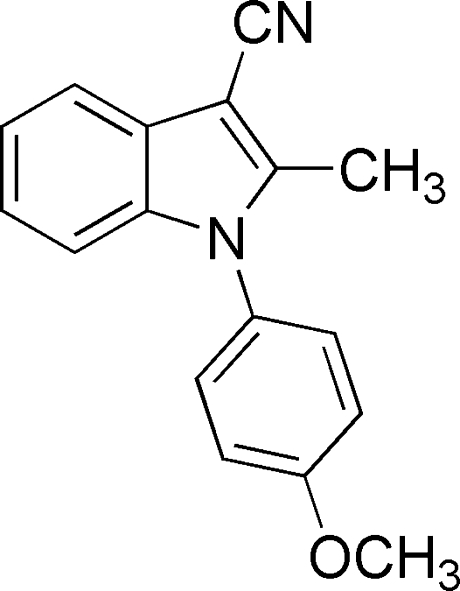


## Experimental

### 

#### Crystal data

C_17_H_14_N_2_O
*M* *_r_* = 262.30
Triclinic, 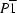

*a* = 7.7381 (10) Å
*b* = 9.4598 (14) Å
*c* = 9.7976 (16) Å
α = 95.983 (2)°
β = 95.464 (4)°
γ = 106.295 (5)°
*V* = 678.79 (17) Å^3^
*Z* = 2
Mo *K*α radiation
μ = 0.08 mm^−1^
*T* = 113 K
0.20 × 0.18 × 0.16 mm

#### Data collection

Rigaku Saturn724 CCD diffractometer
Absorption correction: multi-scan (*CrystalClear*; Rigaku, 2009 ▶) *T*_min_ = 0.984, *T*_max_ = 0.987
8580 measured reflections
3210 independent reflections
2146 reflections with *I* > 2σ(*I*)
*R*_int_ = 0.035

#### Refinement

*R*[*F* ^2^ > 2σ(*F* ^2^)] = 0.035
*wR*(*F* ^2^) = 0.090
*S* = 1.01
3210 reflections
183 parameters
H-atom parameters constrained
Δρ_max_ = 0.19 e Å^−3^
Δρ_min_ = −0.24 e Å^−3^

### 

Data collection: *CrystalClear* (Rigaku, 2009 ▶); cell refinement: *CrystalClear*; data reduction: *CrystalClear*; program(s) used to solve structure: *SHELXS97* (Sheldrick, 2008 ▶); program(s) used to refine structure: *SHELXL97* (Sheldrick, 2008 ▶); molecular graphics: *CrystalStructure* (Rigaku, 2009 ▶); software used to prepare material for publication: *CrystalStructure*.

## Supplementary Material

Crystal structure: contains datablock(s) global, I. DOI: 10.1107/S1600536811031035/hb6329sup1.cif

Structure factors: contains datablock(s) I. DOI: 10.1107/S1600536811031035/hb6329Isup2.hkl

Supplementary material file. DOI: 10.1107/S1600536811031035/hb6329Isup3.cml

Additional supplementary materials:  crystallographic information; 3D view; checkCIF report

## Figures and Tables

**Table 1:** Hydrogen-bond geometry (Å, °) *Cg*2 and *Cg*3 are the centroids of the C2–C7 and C8–C13 rings, respectively.

*D*—H⋯*A*	*D*—H	H⋯*A*	*D*⋯*A*	*D*—H⋯*A*
C3—H3⋯*Cg*3^i^	0.95	2.83	3.6542 (14)	146
C10—H10⋯*Cg*2^ii^	0.95	2.95	3.7133 (14)	138

Symmetry codes: (i) 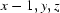; (ii) 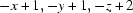.

**Fig. 1.**
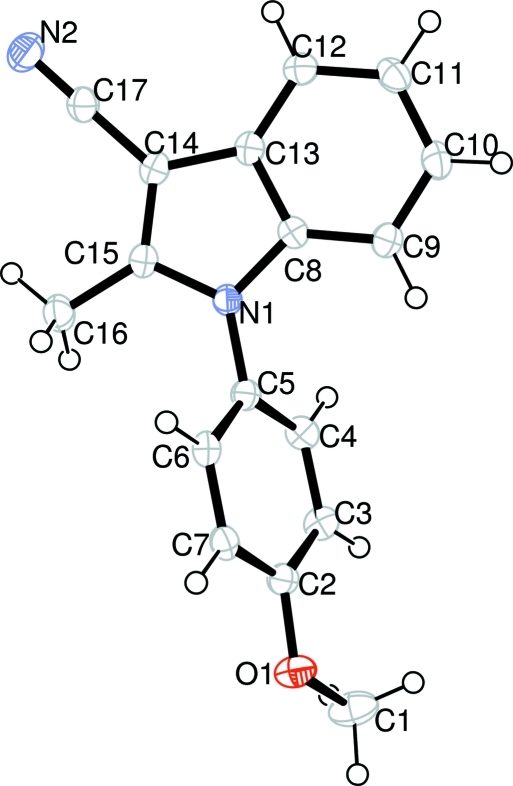
The molecular structure of molecule one of (I) with 50% probability displacement ellipsoids.

## supplementary crystallographic information

### Comment

In the molecular structure of the title compound, (I), (Fig. 1), the indole ring is almost planar with a dihedral angle of 2.66 (6)° between its pyrrole ring and fused benzene ring, which is greater than that [0.85 (6)°] of the 1-(2-chlorophenyl)- 6-fluoro-2-methyl-1*H*-indole-3-carbonitrile reported by Yang *et al.* (2011). The indole ring constructs an angle of 58.41 (4) ° with the methoxylbenzene ring, which is much less than that [80.91 (5)°] reported by Yang *et al.* (2011).

In the crystal packing, π-π stacking interaction and C—H···π interaction help establish the molecular packing. The shortest centroid-centroid separation is 3.8040 (9) Å, which occurs between the benzo part and pyrrole part of the molecules.

### Experimental

The title compound was prepared according to the method of the literature (Du, *et al.*, 2006).  Colourless prisms of (I) were grown from a mixture of ethyl actate and petroleum ether.

### Refinement

All H atoms were positioned geometrically (C—H = 0.95 and 0.98 Å)and refined as riding with *U*_iso_(H) = 1.2*U*_eq_(CH) or 1.5*U*_eq_(CH_3_).

### Crystal data

**Table:**

C_17_H_14_N_2_O	*Z* = 2
*M**_r_* = 262.30	*F*(000) = 276
Triclinic, *P*1	*D*_x_ = 1.283 Mg m^−^^3^
Hall symbol: -P 1	Mo *K*α radiation, λ = 0.71073 Å
*a* = 7.7381 (10) Å	Cell parameters from 2366 reflections
*b* = 9.4598 (14) Å	θ = 2.1–27.9°
*c* = 9.7976 (16) Å	µ = 0.08 mm^−^^1^
α = 95.983 (2)°	*T* = 113 K
β = 95.464 (4)°	Prism, colorless
γ = 106.295 (5)°	0.20 × 0.18 × 0.16 mm
*V* = 678.79 (17) Å^3^	

### Data collection

**Table:**

Rigaku Saturn724 CCD diffractometer	3210 independent reflections
Radiation source: rotating anode	2146 reflections with *I* > 2σ(*I*)
multilayer	*R*_int_ = 0.035
Detector resolution: 14.22 pixels mm^-1^	θ_max_ = 27.9°, θ_min_ = 2.1°
ω and φ scans	*h* = −10→10
Absorption correction: multi-scan (*CrystalClear*; Rigaku, 2009)	*k* = −12→12
*T*_min_ = 0.984, *T*_max_ = 0.987	*l* = −12→12
8580 measured reflections	

### Refinement

**Table:**

Refinement on *F*^2^	Primary atom site location: structure-invariant direct methods
Least-squares matrix: full	Secondary atom site location: difference Fourier map
*R*[*F*^2^ > 2σ(*F*^2^)] = 0.035	Hydrogen site location: inferred from neighbouring sites
*wR*(*F*^2^) = 0.090	H-atom parameters constrained
*S* = 1.01	*w* = 1/[σ^2^(*F*_o_^2^) + (0.038*P*)^2^] where *P* = (*F*_o_^2^ + 2*F*_c_^2^)/3
3210 reflections	(Δ/σ)_max_ = 0.001
183 parameters	Δρ_max_ = 0.19 e Å^−^^3^
0 restraints	Δρ_min_ = −0.24 e Å^−^^3^

### Special details

**Table:**

Geometry. All e.s.d.'s (except the e.s.d. in the dihedral angle between two l.s. planes) are estimated using the full covariance matrix. The cell e.s.d.'s are taken into account individually in the estimation of e.s.d.'s in distances, angles and torsion angles; correlations between e.s.d.'s in cell parameters are only used when they are defined by crystal symmetry. An approximate (isotropic) treatment of cell e.s.d.'s is used for estimating e.s.d.'s involving l.s. planes.
Refinement. Refinement of *F*^2^ against ALL reflections. The weighted *R*-factor *wR* and goodness of fit *S* are based on *F*^2^, conventional *R*-factors *R* are based on *F*, with *F* set to zero for negative *F*^2^. The threshold expression of *F*^2^ > σ(*F*^2^) is used only for calculating *R*-factors(gt) *etc*. and is not relevant to the choice of reflections for refinement. *R*-factors based on *F*^2^ are statistically about twice as large as those based on *F*, and *R*- factors based on ALL data will be even larger.

### Fractional atomic coordinates and isotropic or equivalent isotropic displacement parameters (Å^2^)

**Table:**

	*x*	*y*	*z*	*U*_iso_*/*U*_eq_	
O1	1.23428 (10)	0.89022 (9)	0.06414 (8)	0.0300 (2)	
N1	0.63352 (12)	0.69683 (10)	0.34222 (9)	0.0207 (2)	
N2	0.27094 (14)	0.77814 (11)	0.68887 (10)	0.0350 (3)	
C1	1.34704 (16)	0.79705 (15)	0.03434 (14)	0.0386 (3)	
H1A	1.3990	0.7732	0.1211	0.058*	
H1B	1.4452	0.8492	−0.0148	0.058*	
H1C	1.2741	0.7050	−0.0237	0.058*	
C2	1.08888 (14)	0.83382 (12)	0.13188 (11)	0.0223 (3)	
C3	1.04548 (15)	0.69409 (12)	0.17549 (11)	0.0246 (3)	
H3	1.1173	0.6293	0.1573	0.030*	
C4	0.89572 (14)	0.65052 (12)	0.24598 (11)	0.0241 (3)	
H4	0.8661	0.5558	0.2771	0.029*	
C5	0.78939 (14)	0.74363 (12)	0.27134 (11)	0.0207 (2)	
C6	0.83139 (14)	0.88247 (12)	0.22493 (11)	0.0221 (2)	
H6	0.7570	0.9457	0.2400	0.026*	
C7	0.98146 (14)	0.92716 (12)	0.15712 (11)	0.0226 (2)	
H7	1.0119	1.0225	0.1274	0.027*	
C8	0.48670 (14)	0.57024 (11)	0.29653 (11)	0.0199 (2)	
C9	0.45885 (14)	0.46469 (12)	0.17956 (11)	0.0239 (3)	
H9	0.5482	0.4695	0.1186	0.029*	
C10	0.29541 (15)	0.35277 (13)	0.15628 (12)	0.0279 (3)	
H10	0.2715	0.2794	0.0774	0.033*	
C11	0.16419 (15)	0.34554 (12)	0.24707 (12)	0.0273 (3)	
H11	0.0531	0.2676	0.2283	0.033*	
C12	0.19364 (15)	0.44962 (12)	0.36305 (12)	0.0235 (3)	
H12	0.1051	0.4429	0.4248	0.028*	
C13	0.35650 (14)	0.56517 (12)	0.38782 (11)	0.0205 (2)	
C14	0.42996 (15)	0.69530 (12)	0.48926 (11)	0.0216 (2)	
C15	0.59797 (14)	0.77272 (12)	0.45929 (11)	0.0213 (2)	
C16	0.72912 (15)	0.91064 (12)	0.53687 (11)	0.0293 (3)	
H16A	0.7311	0.9943	0.4852	0.044*	
H16B	0.8506	0.8976	0.5486	0.044*	
H16C	0.6920	0.9310	0.6280	0.044*	
C17	0.34392 (15)	0.74071 (12)	0.60128 (12)	0.0246 (3)	

### Atomic displacement parameters (Å^2^)

**Table:**

	*U*^11^	*U*^22^	*U*^33^	*U*^12^	*U*^13^	*U*^23^
O1	0.0284 (4)	0.0321 (5)	0.0346 (5)	0.0114 (4)	0.0121 (4)	0.0123 (4)
N1	0.0221 (5)	0.0197 (5)	0.0199 (5)	0.0056 (4)	0.0027 (4)	0.0018 (4)
N2	0.0394 (6)	0.0346 (6)	0.0341 (6)	0.0142 (5)	0.0124 (5)	0.0027 (5)
C1	0.0320 (7)	0.0475 (8)	0.0473 (8)	0.0209 (6)	0.0166 (6)	0.0203 (7)
C2	0.0222 (6)	0.0251 (6)	0.0184 (6)	0.0054 (5)	0.0012 (5)	0.0034 (5)
C3	0.0247 (6)	0.0267 (6)	0.0265 (6)	0.0128 (5)	0.0033 (5)	0.0070 (5)
C4	0.0266 (6)	0.0217 (6)	0.0255 (6)	0.0086 (5)	0.0018 (5)	0.0073 (5)
C5	0.0208 (5)	0.0225 (6)	0.0179 (5)	0.0057 (5)	0.0006 (4)	0.0024 (4)
C6	0.0259 (6)	0.0199 (6)	0.0203 (6)	0.0085 (5)	0.0002 (5)	0.0000 (5)
C7	0.0282 (6)	0.0178 (6)	0.0200 (6)	0.0048 (5)	0.0010 (5)	0.0019 (4)
C8	0.0208 (5)	0.0183 (5)	0.0212 (6)	0.0072 (4)	0.0000 (4)	0.0041 (4)
C9	0.0268 (6)	0.0237 (6)	0.0226 (6)	0.0098 (5)	0.0032 (5)	0.0024 (5)
C10	0.0307 (6)	0.0223 (6)	0.0287 (7)	0.0077 (5)	0.0005 (5)	−0.0022 (5)
C11	0.0238 (6)	0.0201 (6)	0.0361 (7)	0.0055 (5)	0.0001 (5)	0.0016 (5)
C12	0.0234 (6)	0.0219 (6)	0.0284 (6)	0.0098 (5)	0.0050 (5)	0.0067 (5)
C13	0.0237 (6)	0.0193 (5)	0.0209 (6)	0.0104 (5)	0.0011 (5)	0.0039 (4)
C14	0.0261 (6)	0.0216 (6)	0.0195 (6)	0.0107 (5)	0.0027 (4)	0.0038 (5)
C15	0.0262 (6)	0.0203 (6)	0.0184 (6)	0.0091 (5)	0.0014 (4)	0.0023 (4)
C16	0.0339 (6)	0.0258 (6)	0.0243 (6)	0.0046 (5)	0.0023 (5)	−0.0006 (5)
C17	0.0279 (6)	0.0213 (6)	0.0258 (6)	0.0090 (5)	0.0028 (5)	0.0040 (5)

### Geometric parameters (Å, °)

**Table:**

O1—C2	1.3675 (12)	C7—H7	0.9500
O1—C1	1.4317 (13)	C8—C9	1.3965 (14)
N1—C15	1.3805 (13)	C8—C13	1.4038 (14)
N1—C8	1.3976 (13)	C9—C10	1.3842 (15)
N1—C5	1.4342 (13)	C9—H9	0.9500
N2—C17	1.1497 (13)	C10—C11	1.4045 (15)
C1—H1A	0.9800	C10—H10	0.9500
C1—H1B	0.9800	C11—C12	1.3799 (15)
C1—H1C	0.9800	C11—H11	0.9500
C2—C7	1.3919 (15)	C12—C13	1.3984 (15)
C2—C3	1.3923 (15)	C12—H12	0.9500
C3—C4	1.3905 (15)	C13—C14	1.4391 (15)
C3—H3	0.9500	C14—C15	1.3770 (15)
C4—C5	1.3837 (14)	C14—C17	1.4261 (15)
C4—H4	0.9500	C15—C16	1.4877 (15)
C5—C6	1.3966 (14)	C16—H16A	0.9800
C6—C7	1.3779 (14)	C16—H16B	0.9800
C6—H6	0.9500	C16—H16C	0.9800
			
C2—O1—C1	117.18 (9)	N1—C8—C13	108.14 (9)
C15—N1—C8	109.22 (9)	C10—C9—C8	117.08 (10)
C15—N1—C5	126.23 (9)	C10—C9—H9	121.5
C8—N1—C5	124.42 (9)	C8—C9—H9	121.5
O1—C1—H1A	109.5	C9—C10—C11	121.28 (10)
O1—C1—H1B	109.5	C9—C10—H10	119.4
H1A—C1—H1B	109.5	C11—C10—H10	119.4
O1—C1—H1C	109.5	C12—C11—C10	121.21 (11)
H1A—C1—H1C	109.5	C12—C11—H11	119.4
H1B—C1—H1C	109.5	C10—C11—H11	119.4
O1—C2—C7	115.32 (10)	C11—C12—C13	118.65 (11)
O1—C2—C3	124.61 (10)	C11—C12—H12	120.7
C7—C2—C3	120.07 (10)	C13—C12—H12	120.7
C4—C3—C2	119.16 (10)	C12—C13—C8	119.40 (10)
C4—C3—H3	120.4	C12—C13—C14	134.75 (10)
C2—C3—H3	120.4	C8—C13—C14	105.80 (10)
C5—C4—C3	120.69 (10)	C15—C14—C17	124.72 (10)
C5—C4—H4	119.7	C15—C14—C13	108.77 (9)
C3—C4—H4	119.7	C17—C14—C13	126.50 (10)
C4—C5—C6	119.91 (10)	C14—C15—N1	108.06 (10)
C4—C5—N1	120.31 (10)	C14—C15—C16	128.79 (10)
C6—C5—N1	119.76 (10)	N1—C15—C16	123.11 (10)
C7—C6—C5	119.59 (10)	C15—C16—H16A	109.5
C7—C6—H6	120.2	C15—C16—H16B	109.5
C5—C6—H6	120.2	H16A—C16—H16B	109.5
C6—C7—C2	120.56 (10)	C15—C16—H16C	109.5
C6—C7—H7	119.7	H16A—C16—H16C	109.5
C2—C7—H7	119.7	H16B—C16—H16C	109.5
C9—C8—N1	129.45 (10)	N2—C17—C14	178.02 (12)
C9—C8—C13	122.36 (10)		
			
C1—O1—C2—C7	−179.16 (9)	C9—C10—C11—C12	−0.16 (17)
C1—O1—C2—C3	1.16 (16)	C10—C11—C12—C13	1.13 (17)
O1—C2—C3—C4	178.73 (9)	C11—C12—C13—C8	−1.51 (16)
C7—C2—C3—C4	−0.94 (16)	C11—C12—C13—C14	175.65 (11)
C2—C3—C4—C5	0.76 (16)	C9—C8—C13—C12	0.98 (16)
C3—C4—C5—C6	0.52 (16)	N1—C8—C13—C12	178.74 (9)
C3—C4—C5—N1	179.03 (9)	C9—C8—C13—C14	−176.92 (9)
C15—N1—C5—C4	125.90 (12)	N1—C8—C13—C14	0.84 (11)
C8—N1—C5—C4	−58.75 (14)	C12—C13—C14—C15	−178.10 (11)
C15—N1—C5—C6	−55.59 (14)	C8—C13—C14—C15	−0.67 (12)
C8—N1—C5—C6	119.76 (11)	C12—C13—C14—C17	0.6 (2)
C4—C5—C6—C7	−1.62 (15)	C8—C13—C14—C17	178.00 (10)
N1—C5—C6—C7	179.86 (9)	C17—C14—C15—N1	−178.46 (10)
C5—C6—C7—C2	1.45 (16)	C13—C14—C15—N1	0.24 (12)
O1—C2—C7—C6	−179.86 (9)	C17—C14—C15—C16	3.84 (18)
C3—C2—C7—C6	−0.16 (16)	C13—C14—C15—C16	−177.46 (10)
C15—N1—C8—C9	176.83 (10)	C8—N1—C15—C14	0.29 (12)
C5—N1—C8—C9	0.80 (17)	C5—N1—C15—C14	176.23 (9)
C15—N1—C8—C13	−0.72 (12)	C8—N1—C15—C16	178.15 (9)
C5—N1—C8—C13	−176.75 (9)	C5—N1—C15—C16	−5.91 (16)
N1—C8—C9—C10	−177.26 (10)	C15—C14—C17—N2	110 (4)
C13—C8—C9—C10	−0.01 (16)	C13—C14—C17—N2	−68 (4)
C8—C9—C10—C11	−0.40 (16)		

### Hydrogen-bond geometry (Å, °)

**Table:**

Cg2 and Cg3 are the centroids of the C2–C7 and C8–C13 rings, respectively.

**Table:**

*D*—H···*A*	*D*—H	H···*A*	*D*···*A*	*D*—H···*A*
C3—H3···Cg3^i^	0.95	2.83	3.6542 (14)	146
C10—H10···Cg2^ii^	0.95	2.95	3.7133 (14)	138

Symmetry codes: (i) *x*−1, *y*, *z*; (ii) −*x*+1, −*y*+1, −*z*+2.
